# Nrf2 Suppression Delays Diabetic Wound Healing Through Sustained Oxidative Stress and Inflammation

**DOI:** 10.3389/fphar.2019.01099

**Published:** 2019-09-20

**Authors:** Min Li, Haibing Yu, Haiyan Pan, Xueqing Zhou, Qiongfang Ruan, Danli Kong, Zhigang Chu, Huawen Li, Jingwen Huang, Xiaodong Huang, Angel Chau, Weiguo Xie, Yuanlin Ding, Paul Yao

**Affiliations:** ^1^Department of General Surgery, Zhongnan Hospital of Wuhan University, Wuhan, China; ^2^Institute of Burns, Tongren Hospital of Wuhan University, Wuhan Third Hospital, Wuhan, China; ^3^School of Public Health, Guangdong Medical College, Dongguan, China; ^4^Key Lab of Prevention and Management of Chronic Kidney Disease of Zhanjiang City, Affiliated Hospital of Guangdong Medical University, Zhanjiang, China

**Keywords:** dimethyl fumarate, HO1, NQO1, Nrf2, wound healing

## Abstract

Impaired wound healing is one of the major complications of diabetes, involving prolonged inflammation, delayed re-epithelialization, and consistent oxidative stress. The detailed mechanism remains unclear, and there is currently no effective treatment for diabetic wound healing. In this study, we aim to investigate the potential role and effect of nuclear factor erythroid-2–related factor-2 (Nrf2) activation on diabetic wound healing. *In vitro* experiments in rat macrophages showed that hyperglycemia treatment suppresses Nrf2 activation, resulting in oxidative stress with decreased expression of antioxidant genes, including NAD(P)H:quinone oxidoreductase 1 and heme oxygenase 1, together with increased secretion of proinflammatory cytokines, including interleukin 1β (IL1β), IL6, and monocyte chemoattractant protein-1. Both Nrf2 overexpression and Nrf2 activator dimethyl fumarate (DMF) treatment significantly ameliorated oxidative stress and inflammation. On the other hand, both Nrf2 knockdown or Nrf2 inhibitor ML385 mimicked the effect of diabetes. Further *in vivo* experiments in rats showed that DMF treatment significantly accelerated wound healing in streptozocin-induced diabetic rats with increased expression of antioxidant enzymes and decreased secretion of proinflammatory cytokines, while Nrf2 inhibitor ML385 mimicked the effect of diabetes. We conclude that Nrf2 activation accelerates impaired wound healing by ameliorating diabetes-mediated oxidative stress and inflammation. This provides a new clinical treatment strategy for diabetic wound healing using Nrf2 activator DMF.

## Introduction

Impaired wound healing is one of the major complications of diabetes. In ∼4% to 10% of patients diagnosed with diabetes mellitus, this may eventually lead to the development of diabetic foot ulcers. These ulcers are a frequent cause of infection, great morbidity, heavy financial burden, and lower extremity amputation (Singh et al., 2005; Noor et al., 2015). Diabetic wounds are characterized by prolonged inflammation with increased inflammatory cytokines (Esposito et al., 2002), delayed re-epithelialization (Thangarajah et al., 2009), and consistent oxidative stress with reactive oxygen species (ROS) overgeneration (El-Osta et al., 2008; McInnes et al., 2014). Taking into account the poor prognosis and the fact that there is still currently no effective therapy for this issue, the detailed mechanism of diabetic wounds still needs to be fully understood and addressed (Kunkemoeller and Kyriakides, 2017; Huang et al., 2019).

Nuclear factor erythroid-2–related factor-2 (Nrf2) is a key transcription factor that regulates the expression of phase II detoxifying enzyme and antioxidant genes (Motohashi and Yamamoto, 2004; da Costa et al., 2019) by responding to pro-oxidative and proinflammatory stress in the cellular defense system (Belcher et al., 2017). Nrf2 maintains a cellular redox balance by regulating many of genes, including, but not limited to, NAD(P)H:quinone oxidoreductase 1 (NQO1) and heme oxygenase 1 (HO1) (Calkins et al., 2009; Dinkova-Kostova and Abramov, 2015). Dimethyl fumarate (DMF) has been reported to activate Nrf2 and is currently used for treatment of psoriasis and multiple sclerosis (Fox et al., 2012), although the potential effect on diabetic wound healing remains largely unknown (Li et al., 2018).

Heme oxygenase 1 is the rate-limiting enzyme that regulates catalytic breakdown of heme into free iron and carbon monoxide (Ahmed et al., 2017). Heme oxygenase 1 is regulated by Nrf2 and plays an important immune-modulatory role in macrophages; the induction of HO1 can switch these cells from the proinflammatory (M1) to anti-inflammatory (M2) phenotype (Luo et al., 2018; Vijayan et al., 2018; Motterlini et al., 2019). It has been reported that HO1 mediates the anti-inflammatory effect through interleukin 10 (IL10) (Lee and Chau, 2002) and IL1β (Hu et al., 2016). Furthermore, the lack of HO1 is associated with increased expression of IL6, IL1β, and monocyte chemoattractant protein-1 (MCP1) (Kozakowska et al., 2018), indicating that Nrf2 may modulate the inflammatory response through HO1 expression in macrophages (Araujo et al., 2012).

In this study, we aim to reveal the potential effect of Nrf2 on diabetic wound healing in macrophages. We found that macrophages in diabetic rats have impaired Nrf2 activity, resulting in increased ROS generation with down-regulation of Nrf2 target genes, including NQO1 and HO1, together with increased secretion of proinflammatory cytokines, including IL1β, IL6, and MCP1. Further investigation showed that either Nrf2 overexpression or Nrf2 activator DMF treatment (Zhu et al., 2018) restores hyperglycemia-induced oxidative stress with up-regulated NQO1 and HO1 as well as decreased secretion of inflammatory cytokines. On the other hand, either Nrf2 knockdown or Nrf2 inhibitor ML385 treatment (Singh et al., 2016) mimicked the effect of hyperglycemia. Further *in vivo* experiments showed that Nrf2 activator DMF significantly accelerates diabetic wound healing in rats with ameliorated oxidative stress and inflammation, while Nrf2 inhibitor ML385 delayed wound healing in regular rats. We conclude that Nrf2 activation accelerates diabetic wound healing through ameliorated oxidative stress and inflammation.

## Materials and Methods

**Materials and Reagents**. Antibodies for β-actin (sc-47778), MafF/G/K (sc-166548), NQO1 (sc-376023), and HO1 (sc-390991) were obtained from Santa Cruz Biotechnology (Shanghai, China). The antibodies for CD31 (ab24590) and Nrf2 (ab137550) were obtained from Abcam (Shanghai, China). 3-Nitrotyrosine was measured by 3-nitrotyrosine ELISA Kit (ab116691 from Abcam). Nuclear extracts were prepared using the NE-PER Nuclear and Cytoplasmic Extraction Reagents Kit (Pierce Biotechnology, Shanghai, China). Protein concentration was measured using the Coomassie Protein Assay Kit (Pierce Biotechnology) per manufacturers’ instructions. Luciferase activity assay was carried out using the Dual-Luciferase™ Assay System (Promega, Shanghai, China), and the transfection efficiency was normalized using a cotransfected renilla plasmid (Zhang et al., 2017). Concanavalin A (#C5275), lipopolysaccharide (LPS) (#L4391), Nrf2 activator DMF (#242926), Nrf2 inhibitor ML385 (#SML1833), and streptozocin (STZ, #S0130) were obtained from Sigma (Shanghai, China).

**Isolation of rat peritoneal macrophages**. Macrophages were isolated from the peritoneal cavity of Wistar rats from 8 to 10 weeks old. A 0.2 ml/ml solution of Concanavalin A was prepared in phosphate-buffered saline (PBS), and 1 ml was injected intraperitoneally into each rat. The rats were anesthetized using isoflurane after 3 days of injection, and a cardiac puncture was conducted to remove as much blood as possible. The abdominal skin was opened, and 10 ml of warm PBS-PS (PBS plus 1% of penicillin and streptomycin) was injected intraperitoneally. After a gentle massage of the abdomen, a small incision was made in the abdominal wall to collect the fluid into a sterile 50 ml conical tube. The abdominal cavity was then rinsed twice with warm PBS-PS, and the collected fluid was centrifuged at 1,000 rpm for 5 min. Sedimentary cells were resuspended with Dulbecco modified eagle medium complete medium (containing 10% fetal bovine serum (FBS), 5 mM glucose, 100 U/ml penicillin, and 100 g/mL streptomycin) and adjusted to a required concentration, and then incubated in 37°C, 5% CO_2_ for 6 hr. Adherent cells were collected and cultured for 18 h followed with 5 ng/ml LPS stimulation for 24 hr for the subsequent experiments (Ray and Dittel, 2010; Yu et al., 2019). Freshly isolated macrophages from diabetic rats showed a morphology similar to that of macrophages that had been isolated from normal rats and given a 4-day high glucose treatment. In this study, we used 4-day high glucose (25 mM)–treated macrophages for the *in vitro* experiments throughout to mimic diabetic conditions of macrophages. Macrophage polarization was evaluated by measurement of mRNA expression of arginase-1 (Arg1) and chitinase 3-like 1 (Ym1) using real-time polymerase chain reaction (PCR) (Naito et al., 2014).

**Construction of NQO1 and HO1 reporter plasmid**. The rat genomic DNA was prepared from rat macrophage cells. In order to construct NQO1/HO1 reporter plasmids, the NQO1 gene promoter (Ensembl gene ID: Nqo1-201 ENSRNOT00000017174.5) and HO1 gene promoter (Ensembl gene ID: Hmox1-201 ENSRNOT00000019192.6) were amplified by PCR and subcloned into the pGL3-basic vector (# E1751, Promega) using indicated restriction sites with the following primers: NQO1 forward: 5’-gcgc-acgcgt- ctt aac cac tga gcc atc tct-3′ (Mlu I) and NQO1 reverse: 5′-gtac-aagctt-ccg cca tgg ctc cag aag ttg-3′ (Hind III); HO1 forward: 5′-gcgc-ggtacc-agttgcgattctgttgtcact-3′ (Kpn I); and HO1 reverse: 5′-gtac-acgcgt-ctg tcg agc tgt ggg cgc tcc-3′ (Mlu I). All the vectors were verified by sequencing, and detailed information on these plasmids is available upon request (Zhang et al., 2017).

**Generation of rat Nrf2/HO1 expression lentivirus**. The rat cDNA for Nrf2 and HO1 (obtained from Open Biosystems, Philadelphia, PA) was subcloned into the pLVX-Puro vector (from Clontech, Beijing, China) with the restriction sites of Xho1 and Xba1 using the below primers: Nrf2 forward primer: 5′-gtac-ctcgag-atg atg gac ttg gaa ttg cca-3′ (Xho1) and Nrf2 reverse primer: 5′-gtac-tctaga-cta gtt ttt ctt tgt atc tgg-3′ (Xba1); HO1 forward primer: 5′-gtac-ctcgag-atg gag cgc cca cag ctc gac-3′ (Xho1) and HO1 reverse primer: 5′-gtac-tctaga-tta cat ggc ata aat tcc cac-3′ (Xba1). The Nrf2, HO1, or empty control (CTL) was expressed through Lenti-X™ Lentiviral Expression Systems (from Clontech) per manufacturers’ instructions (Zou et al., 2017).

**Generation of lentivirus for rat shNrf2/HO1**. The shRNA lentivirus plasmids for rat Nrf2 (sc-156128-SH), HO1 (sc-270124-SH), or nontarget control (sc-108060) were purchased from Santa Cruz Biotechnology. The related lentivirus for Nrf2, HO1, or empty control (CTL) were expressed through Lenti-X™ Lentiviral Expression Systems (from Clontech) per manufacturers’ instructions. The purified and condensed lentiviruses were used for *in vitro* Nrf2 knockdown, and knockdown efficiency was confirmed if mRNA levels were reduced by more than 65% compared to the control group in cells using real-time PCR (**Table S1**).

**Measurement of ROS generation**. Treated cells were seeded in a 24-well plate and incubated with 10μM CM-H2DCFDA (Invitrogen, Shanghai, China) for 45 min at 37°C, and then the intracellular formation of ROS was measured at excitation/emission wavelengths of 485/530nm using a FLx800 microplate fluorescence reader (Bio-Tek, Beijing, China). The data were normalized as arbitrary units (Yao et al., 2005; Zhang et al., 2017).

**RT reaction and real-time quantitative PCR**. Total RNA from treated cells was extracted using the RNeasy Micro Kit (Qiagen, Shanghai, China), and the RNA was reverse transcribed using an Omniscript RT kit (Qiagen). All the primers were designed using Primer 3 Plus software with the Tm at 60°C, primer size of 21 bp, and the product length in the range of 140 to 160 bp (**Table S1**). The primers were validated with an amplification efficiency in the range of 1.9-2.1, and the amplified products were confirmed with agarose gel. The real-time quantitative PCR was run on iCycler iQ (Bio-Rad) with the Quantitect SYBR green PCR kit (Qiagen). The PCR was performed by denaturing at 95°C for 8 min followed by 45 cycles of denaturation at 95°C, annealing at 60°C, and extension at 72°C for 10 s, respectively. One microliter of each cDNA was used to measure target genes. The β-actin was used as the housekeeping gene for transcript normalization, and the mean values were used to calculate relative transcript levels with the ^ΔΔ^CT method per instructions from Qiagen. In brief, the amplified transcripts were quantified by the comparative threshold cycle method using β-actin as a normalizer. Fold changes in gene mRNA expression were calculated as 2^−ΔΔCT^ with CT = threshold cycle, ΔCT = CT (target gene)-CT(β-actin), and the ΔΔCT = ΔCT (experimental)-ΔCT (reference) (Zhang et al., 2017; Zou et al., 2017).

**Immunoprecipitation (IP) and Western blotting (WB)**. Cell lysates were precleared by preimmune IgG plus Protein A agarose beads for 2 h, and the supernatants were immunoprecipitated by the indicated antibodies and a 50% slurry of Protein A agarose beads overnight at 4°C (Metivier et al., 2003). After washing with buffer containing 50 mM Tris, pH 7.5, 150 mM NaCl, 1% NP-40, and 0.5% deoxycholate with protease inhibitors, proteins were released, separated on 10% sodium dodecyl sulfate–polyacrylamide gel electrophoresis gels, blotted by primary antibodies, and then simultaneously incubated with the differentially labeled species-specific secondary antibodies, anti-RABBIT IRDye™ 800CW (green), and anti-MOUSE (or goat) ALEXA680 (red). Membranes were scanned and quantitated by the ODYSSEY Infrared Imaging System (LI-COR, NE) (Ceradini et al., 2008).

**Luciferase reporter assay**. Rat macrophages 1.0 × 10^5^ were seeded in a 6-well plate with complete medium to grow until they reached 80% confluence. Cells were then cotransfected by 3 µg of Vascular endothelial growth factor (VEGF) full length or deletion reporter constructs, together with 0.2 µg of pRL-CMV-Luc *Renilla* plasmid (from Promega). Then, cells were treated by either 5 mM aspirin or empty control (CTL) for 24 h. After treatment, the cells were harvested, and the luciferase activity assays were carried out using the Dual-Luciferase™ Assay System (Promega), and the transfection efficiencies were normalized using a cotransfected *Renilla* plasmid according to manufacturers’ instructions. VEGF reporter activity from different treatments were calculated (Zhang et al., 2017).

**Chromatin immunoprecipitation (ChIP)**. Cells were washed and cross-linked using 1% formaldehyde for 20 min and terminated by 0.1 M glycine. Cell lysates were sonicated and centrifuged. Protein 500 µg was precleared by BSA/salmon sperm DNA with preimmune IgG and a slurry of protein A agarose beads. Immunoprecipitations were performed with the indicated antibodies, BSA/salmon sperm DNA, and a 50% slurry of protein A agarose beads. Input and immunoprecipitates were washed and eluted and then incubated with 0.2 mg/mL proteinase K for 2 h at 42°C, followed by 6 h at 65°C to reverse the formaldehyde cross-linking. DNA fragments were recovered by phenol/chloroform extraction and ethanol precipitation. A ∼150 bp fragment on the NQO1 or HO1 promoter was amplified by real-time quantitative PCR (qPCR) using the primers provided in **Table S1** (Zhang et al., 2017; Zou et al., 2017).

**Enzyme-linked immunosorbent assay**. Rat interleukins from either supernatant or serum, including IL1β, IL6, and MCP1, were measured by Rat IL1β/IL1F2 Quantikine enzyme-linked immunosorbent assay (ELISA) Kit (#RLB00), Rat IL6 Quantikine ELISA Kit (#RRA00), and Rat JE/MCP-1/CCL2 DuoSet ELISA Kit (#DY3144-05), respectively, according to the manufacturer’s instructions from R&D Systems (Kobayashi et al., 2016).

***In vivo* rat experiments**. The animal protocol conformed to US NIH guidelines (Guide for the Care and Use of Laboratory Animals, No. 85-23, revised 1996) and was reviewed and approved by the Institutional Animal Care and Use Committee from Wuhan University. The male Wistar rats were housed four or five per cage on a 12:12-h light-dark cycle and were given commercial rodent chow and water ad libitum on arrival. Chronic diabetic rats were induced by injection of 50 mg/kg STZ (0.05 M sodium citrate, pH 5.5) after an 8-h fasting period at 1 month old. Animals with blood glucose >300 mg/dL were considered positive, while control (CTL) rats received only vehicle injection.

*Rat models of cutaneous burn*. Wild-type and diabetic rats (2 months after STZ injection) were subjected to a model of cutaneous burn injury. The dorsum of each rat was shaved with electric clippers and depilated with Nair. The rats were anesthetized by inhalation of 5% isoflurane, and then the cutaneous burn injury was made on the dorsa of the rats by exposure to a hot copper pillar (2-cm diameter) at 75ºC for 15 s.

*Experimental groups*. The experimental rats were separated into four groups: Group 1. Wild-type (CTL) rats receiving only subcutaneous vehicle (2% DMSO in maize oil) injection; Group 2. Diabetic (STZ) rats received only vehicle injection; Group 3. Diabetic (STZ) rats receiving 15mg/kg/d of DMF (dissolved in DMSO) injection on days of 0, 4, 8, 12, and 16 with respect to the time of wounding (STZ/DMF); Group 4. CTL rats receiving 15 mg/kg/day of ML385 (dissolved in DMSO) injection (CTL/ML385).

*Measurement*. Digital photographs of the wounds were taken every 2 days for 20 days. Wound area was quantified as a percent area of the original wound size using Image J software. At indicated time points, wounds were excised and snap-frozen or, alternatively, processed for hematoxylin-eosin (H&E) staining. Images were taken using a Carl Zeiss MIRAX MIDI slide scanner, and the analyses were performed using a 3DHISTECH Pannoramic Viewer for the quantification of granulation tissue deposition (Zhang et al., 2018). Vascular density was detected on frozen sections by immunohistochemistry using CD31 mouse monoclonal antibody. For quantification of CD31 positivity, wounds were analyzed under 200× magnification, and the numbers of positive six cells per high-power field were counted. All counts and observations were performed by a blinded observer (Thangarajah et al., 2009). Interleukin levels from rat serum were measured using ELISA kits from R&D Systems (Kobayashi et al., 2016). (Naito et al., 2014)Macrophage accumulation was measured by evaluation of N-acetyl-b-d-glucosaminidase (NAG) activity (Canesso et al., 2014). In brief, the tissues were homogenized in 0.9% saline containing 0.1% Triton X-100 (100 mg tissue/1 ml buffer) and then centrifuged at 4°C for 10 min at 3000g, and the supernatants were used for NAG assay. The optical density was measured at 405 nm using NAG (*p*-nitrophenyl-N-acetyl-b-d-glucosaminide, obtained from Sigma). Results were expressed as arbitrary units.

**Immunohistochemistry**. The tissues were dissected and snap-frozen in the OCT compound. The 10-μm sections were cut by clean microtome and mounted on PEN-membrane slides (2.0 μm, Leica) and stored at −20ºC before use. The slides were first fixed by 3.7% formaldehyde at 37ºC for 15 min, permeabilized by 1% BSA+0.2% Triton X-100 in PBS for 1 hour, and then blotted with 40 μg/mL (dilute 1:20) of either NQO1, HO1, or CD31 mouse monoclonal antibody for 2 h. They were then washed three times, and the Texas red–labeled anti-mouse secondary antibody (1:200) was added for blotting for another 1 hour. After thorough washing, the slides were visualized and photographed. The relative densities of each group were quantitated for protein expression using Image J. software (Li et al., 2015).

***In vivo* superoxide release**. Superoxide anion (O_2_^−^) release from the tissue was determined by a luminol-EDTA-Fe enhanced chemiluminescence (CL) system supplemented with dimethyl sulfoxide (DMSO)–tetrabutyl-ammonium chloride solution for extraction of released O_2_^−^ from tissues as described previously (Yao et al., 2005). The superoxide levels were calculated from the standard curve generated by the xanthine/xanthine oxidase reaction (Zhang et al., 2017).

**Statistical analysis**. The data were given as mean ± SEM; all of the experiments were performed at least in quadruplicate unless otherwise indicated. The one-way analysis of variance followed by the Bonferroni *post hoc* test was used to determine statistical significance of different groups. SPSS 22 software was used for statistical analysis, and a *P* value < 0.05 was considered significant (Zhang et al., 2017).

## Results

**Figure 1:**
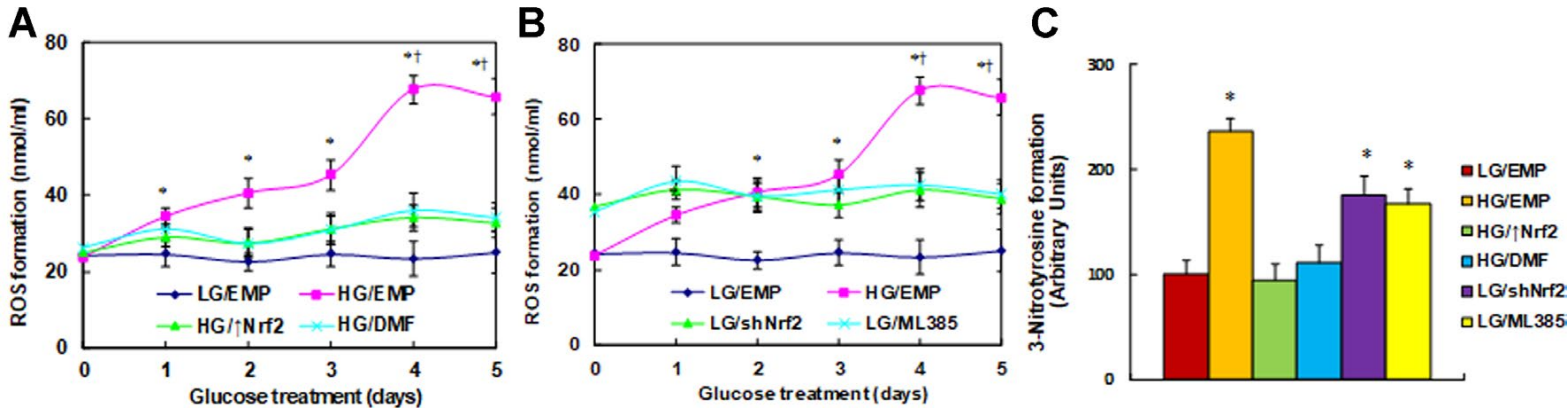
Nrf2 activation restores, while Nrf2 suppression mimics, hyperglycemia-induced ROS generation. **(A)** Rat macrophages were infected by an empty vector (EMP), Nrf2 lentivirus (Nrf2↑), or Nrf2 knockdown lentivirus (shNrf2) in the presence of either 5 µM Nrf2 activator DMF or 5 µM Nrf2 inhibitor ML385 (with 0.1% DMSO as vehicle) and then incubated in either low glucose (5 mM) or high glucose (25 mM glucose) continuously for 5 days in the presence of 1% FBS, and the cells were harvested on each day for analysis. **(A)** ROS generation in the presence of Nrf2 overexpression (Nrf2↑) or Nrf2 activator (DMF), n = 5. *P < 0.05, vs day 0 group; †, *P* < 0.05, vs day 3 group. **(B)** ROS generation in the presence of Nrf2 knockdown (shNrf2) or Nrf2 inhibitor (ML385), n = 5. *P < 0.05, vs day 0 group; ^†^ *P* < 0.05, vs day 3 group. **(C)** 3-Nitrotyrosine formation on day 5 treatment, n = 5. *P < 0.05, vs LG/EMP group. Data were expressed as mean ± SEM.

**Nrf2 activation restores, while Nrf2 suppression mimics, hyperglycemia-induced ROS generation**. We first evaluated the effect of Nrf2 on hyperglycemia-induced ROS generation in macrophages. The cells were treated by high glucose (HG, 25 mM) continuously for 5 days. Reactive oxygen species generation was increased to 145%, 171%, and 190%, respectively, on days 1, 2, and 3, and it further increased to 285% on day 4, reached a plateau compared to the low glucose (LG/EMP) group on day 5, as there was no significant difference from day 4. On the other hand, in the cells that were treated by either Nrf2 expression lentivirus (↑Nrf2) or Nrf2 activator (DMF) in the presence of HG, ROS generation was decreased significantly compared to HG treatment, and it remained constant with no significant increase following the HG treatment (**Figure 1A**). In addition, cells were treated by either Nrf2 knockdown lentivirus (shNrf2) or Nrf2 inhibitor ML385 in the presence of LG. The results showed that ROS generation was increased to 151% compared to the LG/EMP treatment, and it remained constant with no significant changes following the LG treatment (**Figure 1B**). Finally, we measured the 3-nitrotyrosine formation on day 5. The results showed that HG/EMP treatment increased ROS generation to 236% compared to LG/EMP treatment, and the treatment of either Nrf2 expression (HG/↑Nrf2) or Nrf2 activator (HG/DMF) in the presence of HG completely restored the HG effect. On the other hand, treatment of either Nrf2 knockdown (LG/shNrf2) or Nrf2 inhibitor (LG/ML385) in the presence of LG significantly increased ROS generation to 176% and 167%, respectively (**Figure 1C**). Our results indicate that Nrf2 activation restores, while Nrf2 suppression mimics, hyperglycemia-induced ROS generation.

**Figure 2:**
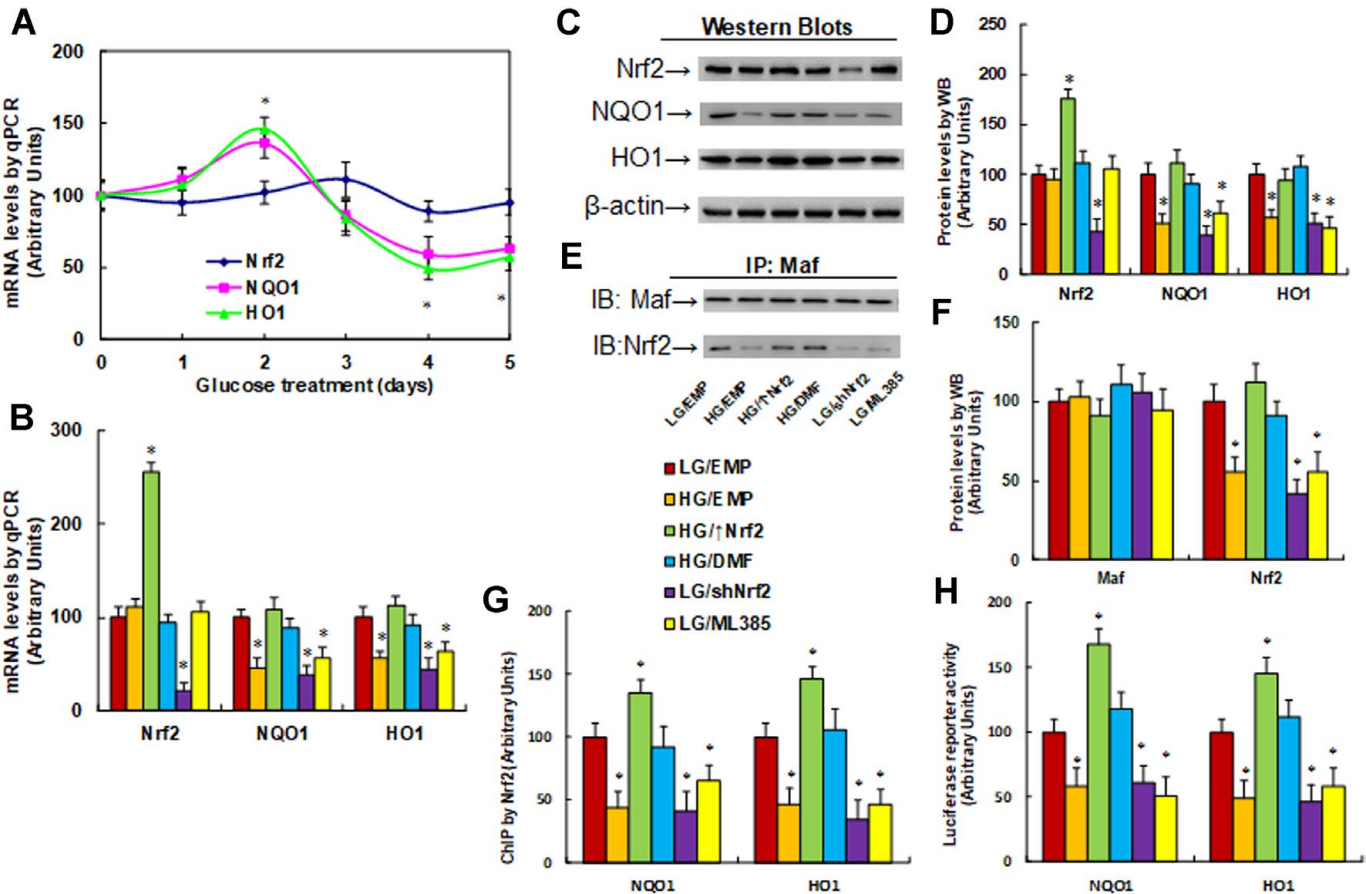
Nrf2 activation increases, while Nrf2 suppression decreases, the expression of NQO1 and HO1. **(A)** Rat macrophages were incubated in hyperglycemia (25 mM glucose) continuously for 5 days in the presence of 1% FBS, and the cells were used for gene analysis, n = 4. *P < 0.05, vs day 0 group. **(B**–**H)** Rat macrophage cells were infected by an empty vector (EMP), Nrf2 lentivirus (Nrf2↑), or Nrf2 knockdown lentivirus (shNrf2) in the presence of either 5 µM Nrf2 activator DMF or 5 µM Nrf2 inhibitor ML385 (with 0.1% DMSO as vehicle), and then incubated in either low glucose (5 mM) or high glucose (25 mM glucose) continuously for 5 days in the presence of 1% FBS. The cells were harvested on day 5 for analysis. *P < 0.05, vs LG/EMP group. **(B)** mRNA analysis by qPCR, n = 4. **(C)** Representative picture for Western blots. **(D)** Protein quantitation for **(C)**, n = 5. **(E)** Representative pictures for IP/WB by Maf/Nrf2. **(F)** Protein quantitation for **(E)**, n = 5. **(G)** ChIP by Nrf2 antibody on NQO1 and HO1 promoter, n = 4. **(H)** Reporter activity assay for NQO1 and HO1, n = 5. Data were expressed as mean ± SEM.

**Nrf2 activation increases, while Nrf2 suppression decreases, the expression of NQO1 and HO1**. We evaluated the potential effect of hyperglycemia on the expression of Nrf2 and its target genes NQO1 and HO1. The macrophages were treated by HG continuously for 5 days, and the mRNA levels were measured. The results showed that Nrf2 mRNA had no significant difference following the HG treatment. On the other hand, the mRNA expression of NQO1 and HO1 increased to 136% and 146%, respectively, on day 2, while it decreased to 59% and 48%, respectively, on day 4, and it remained low as day 4 on day 5. There was no significant difference on days 1 and 3 (**Figure 2A**). Furthermore, the mRNA expression of NQO1 and HO1 showed a biphasic wave with initial up-regulation followed by down-regulation; this can be explained by the inference that Nrf2 was activated during hyperglycemia treatment and subsequent oxidative stress during the first 2 days, while it was eventually suppressed during 4- or 5-day hyperglycemia treatment with posttranslational modification due to long-term oxidative stress (Zhang et al., 2003; Thangarajah et al., 2009). We then measured the mRNA levels on day 5 under different treatments. Nrf2 mRNA levels had no significant difference on treatments of HG, Nrf2 activator DMF, and Nrf2 inhibitor ML385, while Nrf2 expression lentivirus (↑Nrf2) increased, and Nrf2 knockdown lentivirus (shNrf2) decreased Nrf2 mRNA to 256% and 21%, respectively, compared to LG/EMP group, indicating a successful gene manipulation by lentivirus infection. In addition, we found that HG treatment (HG/EMP) decreased expression of NQO1 and HO1 to 46% and 55%, respectively, and Nrf2 expression (HG/↑Nrf2) and Nrf2 activator (HG/DMF) completely restored this effect, while Nrf2 knockdown (LG/shNrf2) and Nrf2 inhibitor (LG/ML385) mimicked the HG effect (**Figure 2B**). We then measured the protein levels of these genes, and an expression pattern similar to that of the mRNA levels was observed (**Figures 2C, D**). We further evaluated the binding ability of Maf protein and Nrf2 in nuclear extracts using IP/WB techniques. The results showed that Maf protein had no significant difference in different treatments, HG treatment (HG/EMP) decreased binding of Maf with Nrf2 to 56% compared to the LG/EMP group, and HG/↑Nrf2 and HG/DMF group completely restored this effect, while LG/shNrf2 and LG/ML385 group mimicked the HG effect (**Figures 2E, F**). We also evaluated the binding ability of Nrf2 on the promoters of NQO1 and HO1 using ChIP techniques. Our findings showed that HG/EMP decreased binding of Nrf2 on the promoter of NQO1 and HO1 to 44% and 46%, respectively, compared to the LG/EMP group, while Nrf2 expression (HG/↑Nrf2) increased the binding ability of Nrf2 to 135% and 147%, respectively. Nrf2 activator (HG/DMF) completely restored the effect of HG, while Nrf2 knockdown (LG/shNrf2) and Nrf2 inhibitor (LG/ML385) mimicked the HG effect (**Figure 2G**). We finally measured the luciferase reporter activity of NQO1 and HO1, and an expression pattern similar to that of Nrf2 binding ability on promoters by ChIP assay was observed (**Figure 2H**). Furthermore, the related target gene activity in **Figures 5G, H** was not consistent or dependent on Nrf2 expression in **Figures 2B, D**; this is because the Nrf2 protein was modified by hyperglycemia-mediated posttranslational modification and lost some activity. Our results indicate that Nrf2 activation increases, while Nrf2 suppression decreases, the expression of NQO1 and HO1.

**Figure 3:**
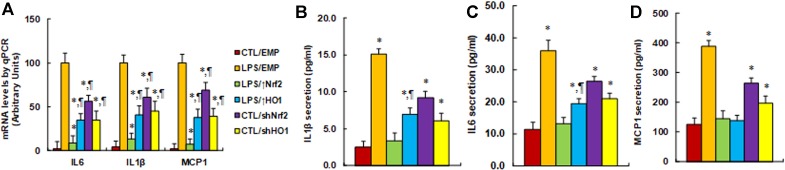
Expression of Nrf2/HO1 prevents, while knockdown of Nrf2/HO1 mimics, hyperglycemia-induced expression of proinflammatory cytokines. Rat macrophages were infected by an empty vector (EMP), Nrf2 lentivirus (Nrf2↑), HO1 lentivirus (HO1↑), Nrf2 knockdown (shNrf2), or HO1 knockdown lentivirus (shHO1) and then incubated in either control (CTL) or 5 ng/mL LPS, and the cells were harvested on day 5 for analysis. **(A)** mRNA levels by qPCR, n = 4. *P < 0.05, vs LPS/EMP group; ¶*P* < 0.05, vs LPS/Nrf2↑ group. **(B)** IL1β secretion, n = 5. *P < 0.05, vs CTL/EMP group; ¶*P* < 0.05, vs LPS/EMP group. **(C)** IL6 secretion, n = 5. *P < 0.05, vs CTL/EMP group; ¶*P* < 0.05, vs LPS/EMP group. **(D)** MCP1 secretion, n = 5. *P < 0.05, vs CTL/EMP group. Data were expressed as mean ± SEM.

**Expression of Nrf2/HO1 prevents, while knockdown of Nrf2/HO1 mimics, hyperglycemia-induced expression of proinflammatory cytokines**. We evaluated the potential effect of Nrf2 and HO1 on the expression of proinflammatory cytokines, including IL1β, IL6, and, MCP1. The macrophages were first stimulated by LPS, then treated by either Nrf2 activation or inhibition, and the expression of those proinflammatory cytokines were analyzed. We first measured the mRNA levels of those cytokines. It showed that LPS treatment (LPS/EMP) significantly increased mRNA levels of IL1β, IL6, and MCP1 by 43, 23, and 56 times, respectively, compared to the CTL/EMP group. Nrf2 expression (↑Nrf2) significantly decreased LPS-induced expression of IL1β, IL6, and MCP1 to 9%, 13%, and 7%, respectively, and HO1 expression (↑HO1) significantly decreased LPS-induced expression of IL1β, IL6, and MCP1 to 35%, 41%, and 38%, respectively, compared to the LPS/EMP group. On the other hand, Nrf2 knockdown (shNrf2) significantly increased basal expression of IL1β, IL6, and MCP1 to 24, 14, and 38 times, respectively, and HO1 knockdown (shHO1) significantly increased basal expression of IL1β, IL6, and MCP1 to 15, 10, and 22 times, respectively, compared to the CTL/EMP group (**Figure 3A**). We then measured the secreted protein levels of those cytokines from cell culture media using ELISA techniques. The results showed that LPS treatment (LPS/EMP) significantly increased secretion of IL1β (**Figure 3B**), IL6 (**Figure 3C**), and MCP1 (**Figure 3D**) by 6.0, 3.2, and 3.1 times, respectively, compared to the CTL/EMP group. Nrf2 expression (LPS/↑Nrf2) completely normalized LPS-induced secretion of those cytokines compared to the LPS/EMP group, while HO1 expression (LPS/↑HO1) showed a much lower effect than Nrf2 expression, as it only partly normalized the effect of LPS. We also evaluated the effect of Nrf2 knockdown (CTL/shNrf2) and HO1 knockdown (CTL/shHO1) on the basal expression of those cytokines. The results showed that gene knockdown of both Nrf2 and HO1 significantly increased the gene expression of those cytokines. Nrf2 knockdown seems have a stronger effect than HO1 knockdown, as it increased basal expression of IL1β, IL6, and MCP1 by 3.7, 2.3, and 2.1 times, respectively, compared to the CTL/EMP group. In addition, we measured the potential effect of Nrf2 and HO1 on macrophage polarization, which was evaluated through the analysis of mRNA expression of Arg1 and Ym1 (**Figure S1**). The results showed that LPS treatment had no effect on the expression of Arg1 and Ym1, while overexpression of Nrf2 (LPS/↑Nrf2) increased mRNA levels of Arg1 and Ym1 to 211% and 196%, respectively; additionally, overexpression of HO1 (LPS/↑HO1) increased mRNA levels of Arg1 and Ym1 to 165% and 158%, respectively, compared to the CTL/EMP group. On the other hand, Nrf2 knockdown (CTL/shNrf2) slightly decreased Arg1 mRNA levels to 65% and had no effect on Ym1 expression. Furthermore, HO1 knockdown (CTL/shHO1) showed no effect on the expression of Arg1 and Ym1. The results indicate that expression of Nrf2 and HO1 have increased macrophage polarization, while knockdown of Nrf2 and HO1 has a small effect.

**Figure 4:**
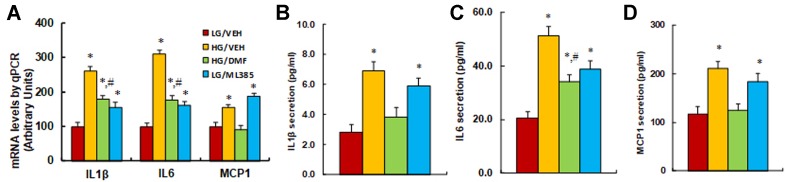
Nrf2 activator DMF prevents, while Nrf2 blocker ML385 mimics, hyperglycemia-induced expression of proinflammatory cytokines. Rat macrophage cells were treated by either 5 µM Nrf2 activator DMF or 5 µM Nrf2 inhibitor ML385, then incubated in either low glucose (5 mM) or high glucose (25 mM glucose) continuously for 5 days, and the cells were harvested on day 5 for further analysis. **(A)** mRNA levels by qPCR, n = 4. **(B)** IL1β secretion, n = 5. **(C)** IL6 secretion, n = 5. **(D)** MCP1 secretion, n = 5. *P < 0.05, vs LG/VEH group; ^#^ *P* < 0.05, vs HG/VEH group. Data were expressed as mean ± SEM.

**Nrf2 activator DMF prevents, while Nrf2 blocker ML385 mimics, hyperglycemia-induced expression of proinflammatory cytokines**. We further evaluated the potential effect of Nrf2 activity on hyperglycemia-induced expression of proinflammatory cytokines. We first measured the mRNA levels of the cytokines; the results showed that high glucose treatment (HG/VEH) significantly increased mRNA expression of IL1β, IL6, and MCP1 to 261%, 311%, and 156%, respectively, compared to the LG/EMP group. Treatment of Nrf2 activator DMF (HG/DMF) partly restored hyperglycemia-induced expression of IL1β and IL6, while it completely restored MCP1 expression compared to the HG/VEH group. In addition, treatment of Nrf2 inhibitor ML385 (LG/ML385) significantly increased the basal expression of IL1β, IL6, and MCP1 to 157%, 161%, and 188%, respectively, compared to the LG/VEH group (**Figure 4A**). We also measured protein secretion in the cell culture media. The results showed that hyperglycemia treatment (HG/VEH) significantly increased the secretion of IL1β (**Figure 4B**), IL6 (**Figure 4C**), and MCP1 (**Figure 4D**) to 261%, 311%, and 156%, respectively, compared to the LG/EMP group. Treatment of Nrf2 activator DMF (HG/DMF) partly restored hyperglycemia-induced IL6 secretion, while it completely restored the secretion of IL1β and MCP1 compared to the HG/VEH group. In addition, treatment of Nrf2 inhibitor ML385 (LG/ML385) significantly increased the basal secretion of IL1β, IL6, and MCP1 to 211%, 146%, and 157%, respectively, compared to the LG/VEH group. Our results indicate that activation of Nrf2/HO1 prevents, while suppression of Nrf2/HO1 mimics, hyperglycemia-induced expression of proinflammatory cytokines.

**Figure 5:**
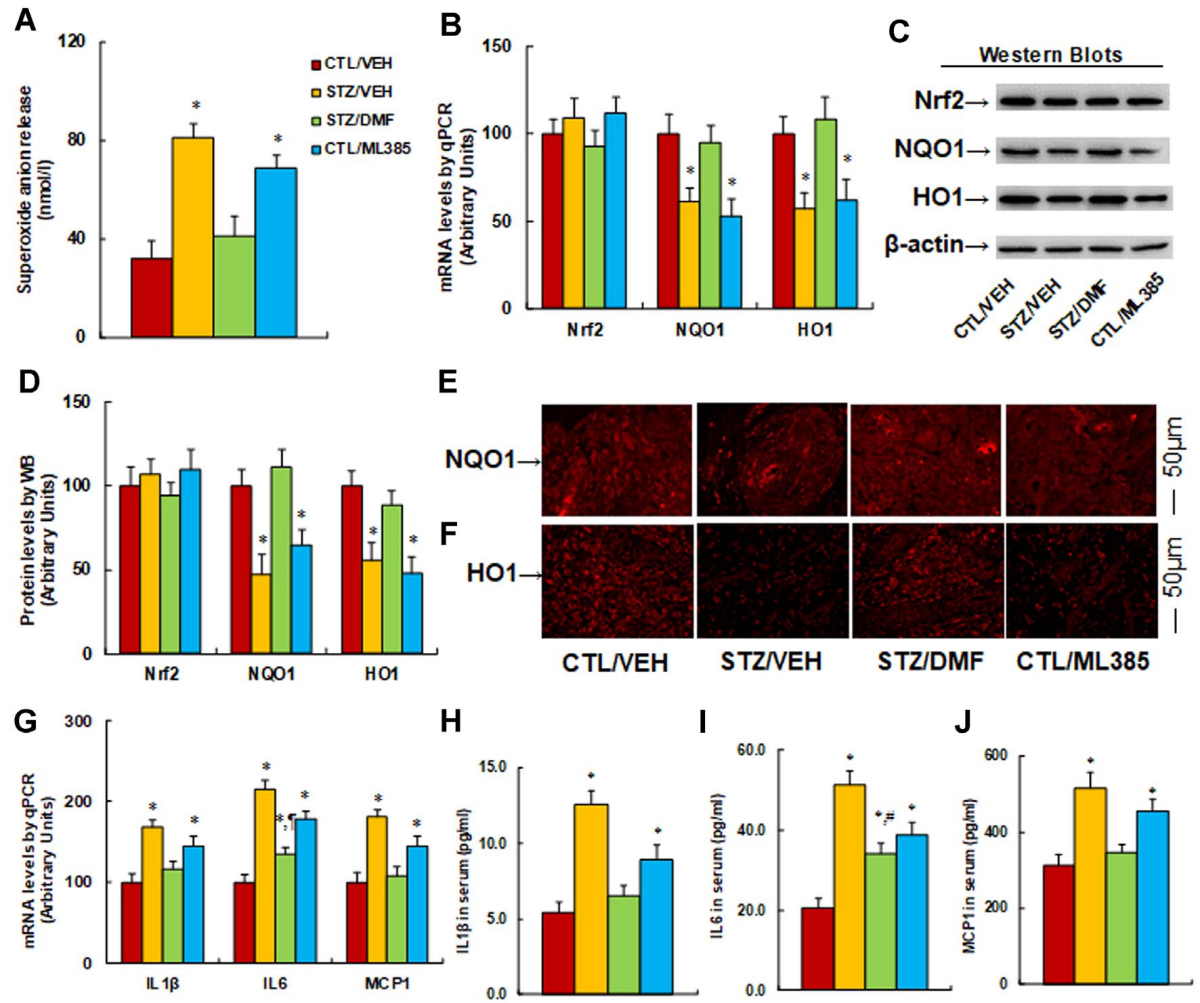
Activation of Nrf2 prevents diabetes-induced oxidative stress and expression of proinflammatory cytokines in rats. The burn injuries were introduced, and the experimental rats were divided into four groups: control rats (CTL/VEH), STZ diabetic rats (STZ/VEH), STZ rats treated by 15 mg/kg/d of DMF (STZ/DMF), or CTL rats received 15 mg/kg/d of ML385 (CTL/ML385). The wounds were excised and collected from the experimental rats for further analysis. **(A)** Superoxide anion release, n = 5. **(B)** mRNA levels for Nrf2 and its target genes by qPCR, n = 4. **(C)** Representative pictures for Western blots. **(D)** Protein quantitation for **(C)**, n = 4. **(E)** Representative pictures for NQO1 expression by immunohistochemistry. **(F)** Representative pictures for HO1 expression by immunohistochemistry. **(G)** mRNA levels for proinflammatory cytokines, n = 4. **(H)** IL1β in serum, n = 5. **(I)** IL6 in serum, n = 5. **(J)** MCP1 in serum, n = 5. *P < 0.05, vs CTL group; ¶*P* < 0.05, vs STZ/VEH group. Data were expressed as mean ± SEM.

**Activation of Nrf2 prevents diabetes-induced oxidative stress and expression of proinflammatory cytokines**. The burn injuries were introduced, and the experimental rats were divided into four groups: control rats (CTL/VEH), STZ diabetic rats (STZ/VEH), STZ rats treated by 15 mg/kg/d of DMF (STZ/DMF), or CTL rats receiving 15 mg/kg/d of ML385 (CTL/ML385), for further analysis. The wounds were excised and collected from the experimental rats for further analysis on day 6 after the introduction of burn injury. We first evaluated the potential effect of Nrf2 activation on diabetes-induced oxidative stress in wound tissues. The results showed that diabetic rats (STZ/VEH) significantly increased superoxide anion (O_2_^−^) release to 253%, Nrf2 activator DMF (STZ/DMF) treatment completely normalized this effect, and Nrf2 inhibitor in control rats (CTL/ML385) increased O_2_^−^ release to 216% compared to control (CTL/VEH) group, mimicking the effect of diabetes (**Figure 5A**). We then measured mRNA levels for Nrf2 and its target genes. The results showed that Nrf2 had no change in different treatments, while diabetic rats (STZ/VEH) decreased mRNA levels of NQO1 and HO1 to 61% and 57%, respectively. Nrf2 activation (STZ/DMF) completely restored this effect, while Nrf2 inhibitor (CTL/ML385) decreased mRNA levels of NQO1 and HO1 to 53% and 62%, respectively, compared to the control (CTL/VEH) group (**Figure 5B**). We also measured the protein levels for those genes, and an expression pattern similar to that of the mRNA levels was observed (**Figures 5C, D**). We also evaluated the expression of NQO1 (**Figure 5E**) and HO1 (**Figure 5F**) using immunohistochemistry techniques; the results showed expression levels consistent with protein levels. Furthermore, we evaluated the potential effect of Nrf2 activation on diabetes-induced proinflammatory cytokines. We found that diabetic rats (STZ/VEH) increased mRNA levels of IL1β, IL6, and MCP1 to 168%, 215%, and 181%, respectively. Nrf2 activation (STZ/DMF) completely restored the expression of IL1β and MCP1 but only partly restored IL6 expression. On the other hand, Nrf2 inhibitor (CTL/ML385) increased mRNA levels of IL1β, IL6, and MCP1 to 145%, 178%, and 146%, respectively, compared to the control (CTL/VEH) group (**Figure 5G**). We then measured the serum levels of those proinflammatory cytokines, including IL1β (**Figure 5H**), IL6 (**Figure 5I**), and MCP1 (**Figure 5J**), and an expression pattern similar to that of mRNA levels was observed. Our results indicate that activation of Nrf2 prevents diabetes-induced oxidative stress and expression of proinflammatory cytokines.

**Figure 6:**
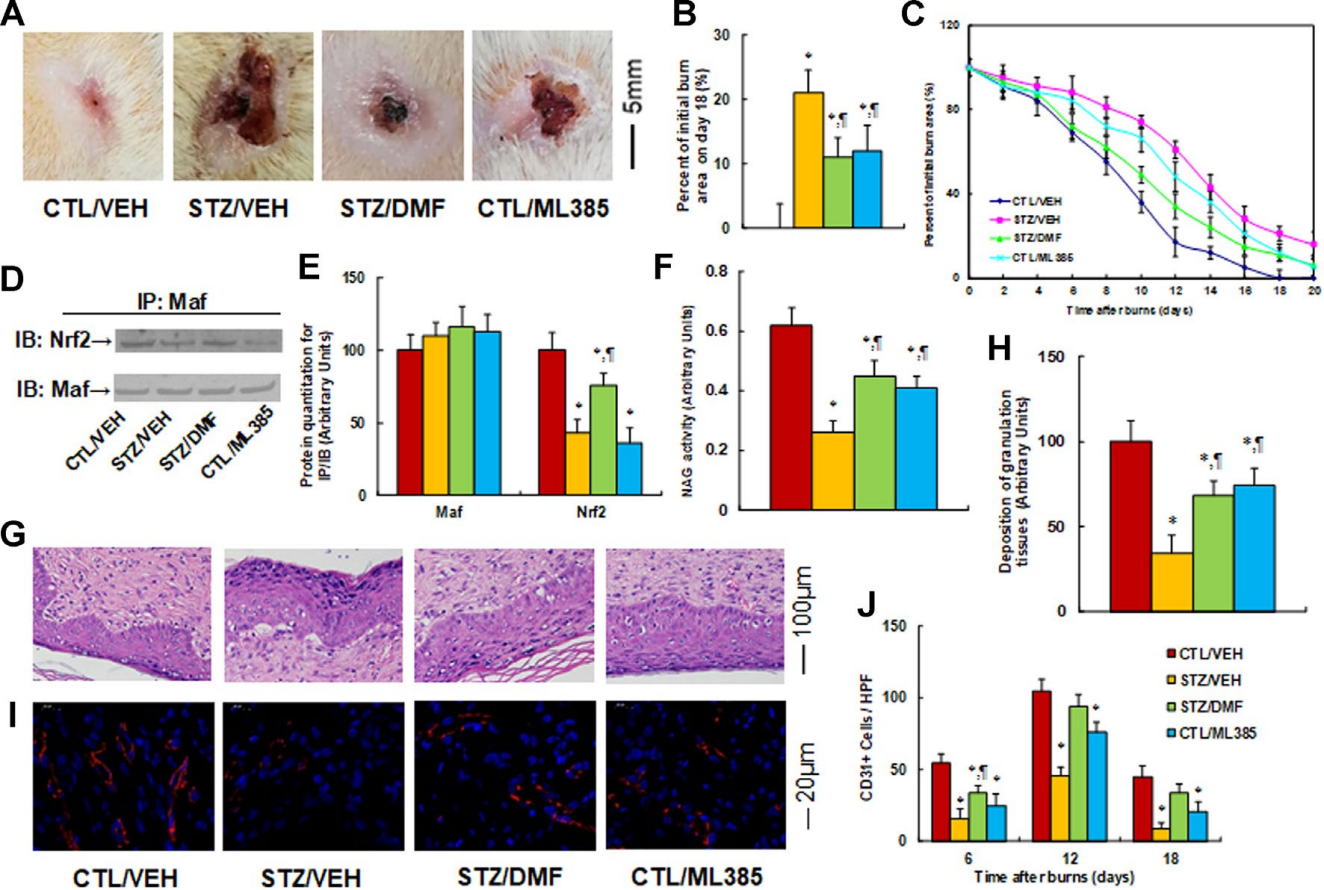
Nrf2 activation accelerates wound healing in diabetic rats, while Nrf2 inhibitor mimics the effect of diabetes. The experimental rats were divided into four groups: control rats (CTL/VEH), STZ diabetic rats (STZ/VEH), STZ rats treated by 15 mg/kg/d of DMF (STZ/DMF), or CTL rats received 15 mg/kg/d of ML385 (CTL/ML385), for further analysis. **(A)** Photographs of representative wounds on day 12 after burns. **(B)** Quantitation of burn area on day 18, n = 8. **(C)** Graphical depiction of wound areas on different days after burns, n = 8. **(D)** Representative pictures for IP/WB by Maf/Nrf2. **(E)** Protein quantitation for **(D)**, n = 5. **(F)** NAG activity as the marker of macrophage accumulation, n = 5. **(G)** Hematoxylin-eosin stains of wound tissues on day 12 after burns with occurrence of granulation tissue in the wounds. **(H)** Deposition of granulation tissues, n = 8. **(I)** The representative pictures for evaluation of vascularity (assessed by CD31 immunohistochemistry) on day 12 after burns. **(J)** Numbers of CD31 positive vessels per high-power field area on day 12 after burns, n = 8. *P < 0.05, vs CTL/VEH group; ¶*P* < 0.05, vs STZ/VEH group. Data were expressed as mean ± SEM.

**Nrf2 activation accelerates wound healing in diabetic rats, while Nrf2 inhibitor mimics the effect of diabetes**. We evaluated the possible effect of Nrf2 activation on wound healing during burn injury in rats. The burn injury was introduced in either control (CTL/VEH) or diabetic (STZ/VEH) rats and then treated by either Nrf2 activator (STZ/DMF) or Nrf2 inhibitor (CTL/ML385), and then the healing process of the burn injuries was evaluated. We first measured the wound healing rate on different time points with different treatments. The results showed that diabetes (STZ/VEH) delayed wound healing compared to the control (CTL/VEH) group, and Nrf2 activator (STZ/DMF) significantly accelerated wound healing, while Nrf2 inhibitor (CTL/ML385) mimicked the diabetic effect (**Figures 6A–C**). **Figure 6A** shows representative pictures of the wound areas on day 12, and **Figure 6B** shows the percentage of burn area on day 18. The diabetic (STZ/VEH) group had significantly higher burn area compared to the completely recovered control (CTL/VEH) group, while both the STZ/DMF or CTL/ML385 groups showed less burn area than the STZ/VEH group, but did not recover completely. **Figure 6C** indicates the quantitated relative wound areas following different time points. We then measured Nrf2 activity under different treatments by evaluating the interaction of Maf and Nrf2 using IP techniques (**Figures 6D, E**). The results showed that diabetes (STZ/CTL) decreased interaction of Nrf2 with Maf to 43% compared to the control (CTL/VEH) group; Nrf2 activator (STZ/DMF) partly restored this effect, while Nrf2 inhibitor (CTL/ML385) mimicked the effect of diabetes. We also measured macrophage accumulation by evaluating NAG activity (**Figure 6F**). The results showed that diabetes (STZ/CTL) decreased NAG activity to 42% compared to the control (CTL/VEH) group; Nrf2 activator (STZ/DMF) partly restored this effect, while Nrf2 inhibitor (CTL/ML385) partly mimicked the effect of diabetes. We also measured the deposition of granulation tissues using H&E staining on day 12 after the burn injuries (**Figures 6G, H**). The results showed that in diabetic rats (STZ/VEH) deposition of granulation tissues decreased to 34%, while this effect was significantly restored to 68% in the Nrf2 activator (STZ/DMF) group compared to the control (CTL/VEH) group; the Nrf2 inhibitor (CTL/ML385) group partly mimicked the effect of diabetes in the rats. In addition, we evaluated neovascularization through immunohistochemistry staining of CD31 positive (CD31^+^) cells (**Figures 6I, J**). We found that the number of CD31^+^ cells decreased to 29%, 44%, and 20%, respectively, on days 6, 12, and 18 in diabetic rats (STZ/VEH) compared to control (CTL/VEH) rats; this effect was partly restored by Nrf2 activator DMF (STZ/DMF), while Nrf2 inhibitor (CTL/ML385) mimicked the effect of diabetes. Our results indicate that Nrf2 activation accelerates wound healing in diabetic rats, while Nrf2 inhibitor mimics the effect of diabetes.

## Discussion

In this study, we demonstrated that macrophages in diabetic rats have sustained oxidative stress and inflammation due to impaired Nrf2 activity-mediated downregulation of Nrf2 target genes (including NQO1 and HO1) and increased inflammatory cytokines (including IL1β, IL6, and MCP1). Nrf2 activation by DMF ameliorated Nrf2 suppression-mediated oxidative stress and inflammation and subsequently accelerated diabetic wound healing. This provides a potential therapeutic strategy for diabetic wound healing through Nrf2 activation.

**Hyperglycemia-induced Nrf2 suppression and oxidative stress**. We showed that hyperglycemia treatment induces Nrf2 suppression with oxidative stress and down-regulated Nrf2 target genes, while Nrf2 expression, including both mRNA and protein levels, had no change. Further investigation showed that hyperglycemia treatment decreases the ability of Nrf2 to bind with the Maf protein in the nucleus, and subsequently results in Nrf2 suppression and down-regulation of Nrf2 target genes (Motohashi et al., 2004). Our results indicate a posttranslational modification of Nrf2 mediated by hyperglycemia-induced oxidative stress was involved in this process. This may include, but not be limited to, Nrf2 phosphorylation (Bloom and Jaiswal, 2003), methylglyoxal modification (Thangarajah et al., 2009), O-GlcNAc modification (Zhang et al., 2003), and ubiquitin-proteasome degradation (Sekhar et al., 2002). In this study, we showed that hyperglycemia suppresses Nrf2 activity and its target antioxidant genes, subsequently resulting in oxidative stress. Nrf2 regulates many of the antioxidant genes, including NQO1, mitochondrial superoxide dismutase (SOD2), glutathione S-transferase, glutamylcysteine synthetase, and so on. (Jaiswal, 2004; Motohashi and Yamamoto, 2004). In this study, NQO1 was selected as one of the representative Nrf2-regulated antioxidant genes. Overexpression of NQO1 can partly restore Nrf2 suppression-mediated oxidative stress in hyperglycemia or diabetic conditions, but cannot completely restore this effect. For instance, it cannot diminish ROS generation in the mitochondria; instead, SOD2 plays an important role in diminishing mitochondria-derived ROS generation. In addition, overexpression of both NQO1 and HO1 cannot fully mimic the action of Nrf2 in macrophages.

**HO1-mediated inflammation in diabetes**. We showed that HO1 plays an important role in the regulation of hyperglycemia-mediated inflammatory cytokines. Heme oxygenase 1 overexpression suppresses hyperglycemia-induced gene expression of IL1β, IL6, and MCP1. On the other hand, HO1 knockdown mimics the effect of hyperglycemia, increasing the gene expression of those cytokines. It has been reported that HO1 plays a major immunomodulatory role in both *in vivo* (Poss and Tonegawa, 1997; Yachie et al., 1999) and *in vitro* cell culture (Kapturczak et al., 2004; Greil et al., 2016), and our results are consistent with these previous findings. There is no evidence showing direct regulation of HO1 on the expression of those proinflammatory cytokines. On the other hand, HO1 may indirectly modulate the gene expression of those cytokines, including IL6, IL1β, and MCP1 (Vijayan et al., 2018; Lee and Chau, 2002; Hu et al., 2016; Kozakowska et al., 2018).

**Role of Nrf2 activation in diabetic wound healing**. We showed that Nrf2 activity was suppressed in hyperglycemia with subsequent oxidative stress and inflammation, while there was no change in gene expression. On the other hand, Nrf2 activation by DMF treatment restored this effect and significantly accelerated diabetic wound healing. The DMF modifies cysteine (Cys) residue on the KEAP1 (Kelch-like ECH associated protein 1) protein, and the modification of KEAP1 then dissociates with Nrf2. The subsequent Nrf2 translocation and binding with the Maf protein initiate the transcription of antioxidant genes and the cellular anti-inflammatory responses (Calkins et al., 2009; Schulze-Topphoff et al., 2016; Zhu et al., 2018). In addition, the activation of Nrf2 may also promote mitochondrial biogenesis by positively regulating nuclear respiratory factor 1 and indirectly ameliorate hyperglycemia-induced oxidative stress (Hayashi et al., 2017).

## Conclusions

Hyperglycemia treatment suppresses Nrf2 activation with subsequent oxidative stress and inflammation. Either Nrf2 overexpression or Nrf2 activation by DMF treatment ameliorates the effect of hyperglycemia in rat macrophages. On the other hand, either Nrf2 knockdown or Nrf2 inhibitor ML385 mimics the effect of hyperglycemia. The *in vivo* rat experiments show that Nrf2 activator DMF accelerates diabetic wound healing with ameliorated oxidative stress and decreased expression of proinflammatory cytokines, including IL1β, IL6, and MCP1. We conclude that Nrf2 activation accelerates diabetic wound healing by ameliorating oxidative stress and inflammation, providing a new clinical therapeutic strategy for diabetic wound healing using Nrf2 activator DMF.

## Data Availability

All datasets generated for this study are included in the manuscript/**Supplementary Files**.

## Ethics Statement

The animal protocol conformed to US NIH Guidelines (Guide for the Care and Use of Laboratory Animals, No. 85-23, Revised 1996) and was reviewed and approved by the Institutional Animal Care and Use Committee from Wuhan University.

## Author Contributions

PY wrote the paper. PY, YD, and WX designed, analyzed the data, and interpreted the experiments. HP, DK, and HL performed vector constructions and gene expression analysis. XZ, QR, ZC, JH, XH, and AC performed statistical analysis and part of the rat experiments. ML and HY performed the remaining experiments. All authors read and approved the final manuscript.

## Funding

This study was financially supported by The National Natural Science Foundation of China, Project #: 81772097 & 81501667; Hubei Science & Technology Development Project #: 2016CFB473 & 2016CFB589; The Features Innovative Projects of General Colleges and Universities of Guangdong Province in 2017 (NO.2017KTSCX084); The Doctoral Research Project of Guangdong Natural Science in 2018 (Basic and Applied Basic Research (2018A030310115); and Open Funding of Key Lab of Prevention and Management of Chronic Kidney Disease of Zhanjiang City, Project #: KF201905.

## Conflict of Interest Statement

The authors declare that the research was conducted in the absence of any commercial or financial relationships that could be construed as a potential conflict of interest.

## Abbreviations

Arg1, arginase-1; ChIP, chromatin immunoprecipitation; DMF, dimethyl furamate; HO1, heme oxygenase 1; IL6, interleukin-6; IL1β, interleukin-1β; NQO1, NAD(P)H quinone dehydrogenase 1; LPS, lipopolysaccharide; MCP1, monocyte chemoattractant protein-1; Nrf2, nuclear factor erythroid-2–related factor-2; O_2_^−^, superoxide anion; qPCR, quantitative real-time PCR; ROS, reactive oxygen species; STZ, streptozotocin; Ym1, chitinase 3-like 1.
