# Supplementary material for: Nrf2 Suppression Delays Diabetic Wound Healing Through Sustained Oxidative Stress and Inflammation
Source: Front Pharmacol. 2019 Sep 20;10:1099. doi: 10.3389/fphar.2019.01099 (PMC6763603; doi:10.3389/fphar.2019.01099)
Supplement: Supplementary file 1 [file Table_1.docx]

**Nrf2 Suppression Delays Diabetic Wound Healing through Sustained Oxidative Stress and Inflammation**

Min Li^1,2†^, Haibing Yu^3,4†^, Haiyan Pan^3^, Xueqing Zhou^2^, Qiongfang Ruan^2^, Danli Kong^3^, Zhigang Chu^2^, Huawen Li^3^, Jingwen Huang^2^, Xiaodong Huang^2^, Angel Chau^2^,

Weiguo Xie^2*^, Yuanlin Ding^3*^, Paul Yao^2,3*^

**Supplemental Information**

**Table S1. Sequences of primers for the real time quantitative PCR (qPCR)**

| Gene | Species | Analysis | Forward primer (5'→3') | Reverse primer (5'→3') |
| --- | --- | --- | --- | --- |
| HO1 | Rat | ChIP | tcctgccttcctatcactgaa | ctggcctgtatggggataaat |
| NQO1 | Rat | ChIP | agatcttggacagggagcagt | aagttattggcagggagaagc |
| β-actin | Rat | mRNA | aggtcatcactatcggcaatg | gcactgtgttggcatagaggt |
| HO1 | Rat | mRNA | caagcagaacccagtctatgc | cgctttacgtagtgctgtgtg |
| MCP1 | Rat | mRNA | tcacctgctgctactcattca | attccttattggggtcagcac |
| NQO1 | Rat | mRNA | cattctgaaaggctggtttga | ccctgcagagagtacatggag |
| Nrf2 | Rat | mRNA | cagcttttggcagagacattc | taaatcagtcatggccgtctc |
| IL1β | Rat | mRNA | gagagtgtggatcccaaacaa | ggaagacaggtctgtgctctg |
| IL6 | Rat | mRNA | agccagagtcattcagagcaa | gtcttggtccttagccactcc |
| Arg1 | Rat | mRNA | tggactggacccagtattcac | gtcttcccaagagttgggttc |
| Ym1 | Rat | mRNA | tatgatgttgcccagatagcc | tccacattgctgaatctgtca |

FIGURE S1

**Figure 1s.** **Expression of** **Nrf2/HO1 activates macrophage polarization.** Rat macrophages were infected by an empty vector (EMP), Nrf2 lentivirus (Nrf2↑), HO1 lentivirus (HO1↑), Nrf2 knockdown (shNrf2) or HO1 knockdown lentivirus (shHO1), then incubated in either control (CTL) or 5ng/ml LPS, and the cells were harvested on day 5 to measure the mRNA expression of Arg1 and Ym1 as the macrophage polarization marker. n=5. *, *P*<0.05, vs CTL/EMP group. Data were expressed as mean ± SEM.
